# Typical trident sign and cardiac involvement in a patient suspected to Sarcoidosis despite negative whole-body FDG-PET: a case report

**DOI:** 10.1186/s13256-023-04224-1

**Published:** 2023-11-30

**Authors:** Abootorab Shahmohammdi, Hora Heidari, Kosar Kohandel, Soheil Dousti, Rozita Doosti, Amir Reza Azimi, Zahra Shajari, Parham Rabiei, Sareh Shahmohammdi

**Affiliations:** 1 Multiple Sclerosis Research Center, Neuroscience Institute, Tehran University of Medical Sciences, Sina Hospital, Hasan Abad Square, Tehran, Iran; 2 Cardiovascular disease Department, School of Medicine, Tehran University of Medical Sciences, Tehran, Iran; 3 Rajaei Cardiovascular and Medical Research Center, Iran University of Medical Sciences, Tehran, Iran

**Keywords:** Neuro-sarcoidosis, Trident sign, FDG-PET scan, Endomyocardial biopsy, Cardiac sarcoidosis, Case report

## Abstract

**Background:**

Sarcoidosis is a systemic inflammatory disease histologically defined by the non-caseation granulomas formation in different organs, most commonly lungs, liver, skin, gastrointestinal system, eyes, neurologic and cardiac system

**Case presentation:**

We report the case of a 42-year-old Gilaks woman who presented with myelopathy with characteristic MRI finding called trident sign. By finding this view in axial spinal Magnetic Resonance Imaging (MRI) imaging, a systemic evaluation was performed on the patient, which led to the diagnosis of cardiac involvement in Sarcoidosis with the specific appearance of this disease in cardiac MRI despite the negative Fluorodeoxyglucose (FDG)-positron emission tomography (PET) scan.

**Conclusions:**

Sometimes characteristic findings such as the trident sign prompt the physician to high suspicion and wide evaluation of the patient to reveal important organ involvement that changes the treatment decision and saves the patient.

## Background

Sarcoidosis is a systemic inflammatory disease histologically defined by the non-caseation granulomas formation of subsequent tissue scarring in different organs, most commonly lungs, liver, skin, gastrointestinal system, eyes, neurologic and cardiac system. According to the diagnostic criteria of neuro-sarcoidosis (NS), pathologic confirmation in the nervous system is needed for a definitive diagnosis and patients with a probable diagnosis need a systemic pathologic confirmation (tissue outside the nervous system). For a possible diagnosis in the absence of pathological findings, we need a set of findings that highly suggest the disease on imaging or other para-clinical tests [1]. Although the gold standard for diagnosing sarcoidosis is a tissue biopsy of the involved organ, but there are three guidelines for the diagnosis of Cardiac Sarcoidosis (CS) to allow for diagnosis confirmation without biopsy including: 1- 2014 Heart Rhythm Society (HRS). 2- World Association of Sarcoidosis and Other Granulomatous Disorders Sarcoidosis Organ (WASOG). 3- Japanese Ministry of Health & Welfare (JMHW) guidelines with 2017 [2]

Considering the difficulty of finding proper and adequate tissue for biopsy in the heart and brain, identifying specific findings in non-invasive methods such as MRI can be very helpful in distinguishing this disease from other differential diagnoses [3].

## Case presentation

A 42-year-old Gilaks female patient presented with mild lower limb weakness. She had hypokalemia in the initial lab tests, so the patient's weakness was attributed to hypokalemia in several visits and finally, she was diagnosed with renal tubular acidosis.

**Fig. 1:**
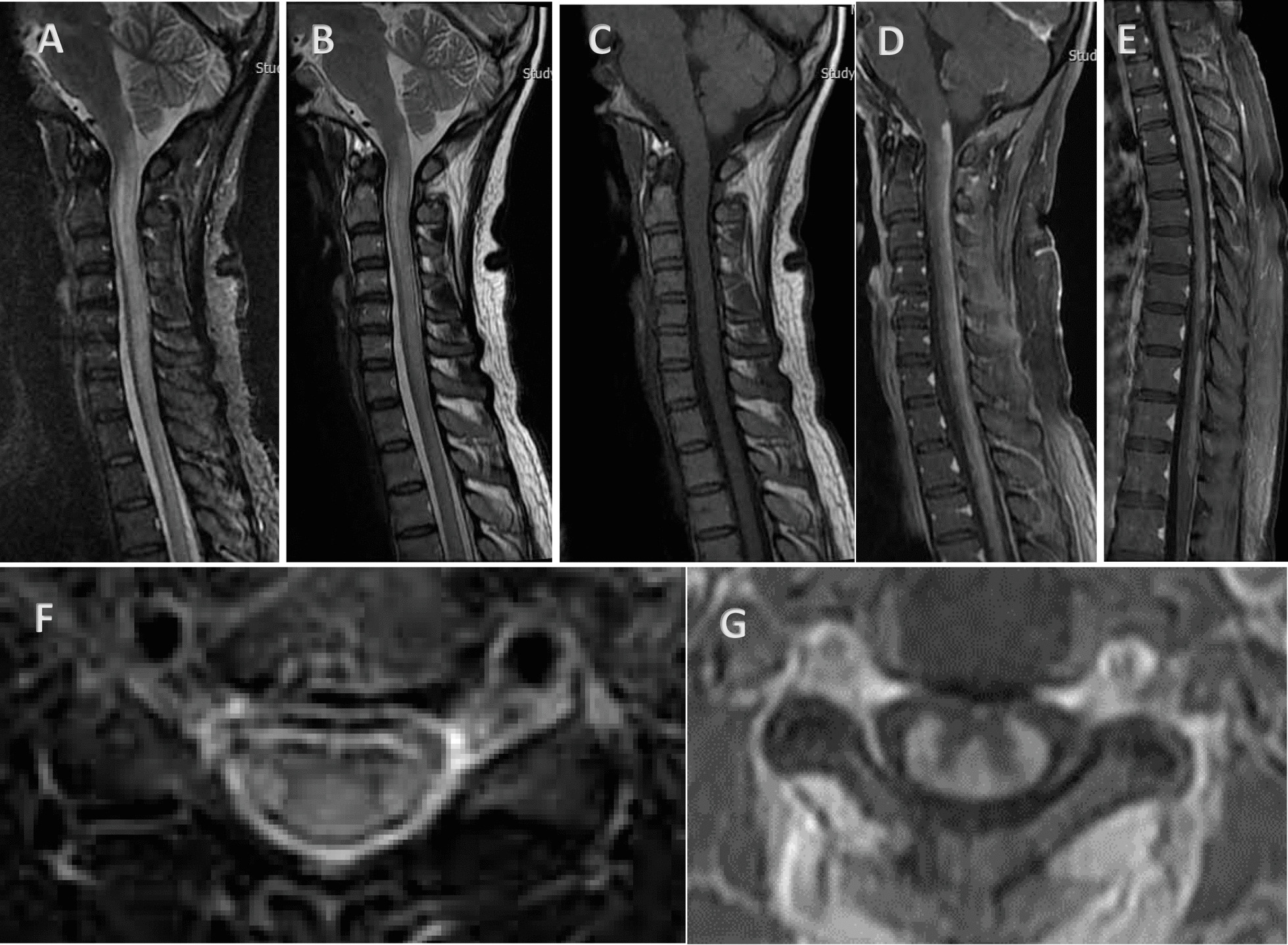
**A**, **B** hyperintense lesion in STIR and T2 weighted images as a LETM. **C** isointense in T1 weighted image. **D**, **E** dorsal subpial longitudinal enhancement in T1 post contrast. **F**, **G** trident sign on axial T2 weighted and T1 post contrast images. *LETM* Longitudinally Extensive Transverse Myelitis. *STIR* Short Tau Inversion Recovery

After three months, the patient's weakness increased and the symptoms of urinary frequency and urgency along with dysesthesia and pain were added. In the examination, she had progressive spastic paraparesis with hyperreflexia and impaired position sense. She was able to walk with help. After brain and cervical Magnetic Resonance Imaging (MRI) without gadolinium, she was admitted and treated with steroid pulse (1000 mg methylprednisolone) with the diagnosis of longitudinally extensive transverse myelitis (LETM) with normal brain MRI. AQP4 antibody test was borderline by enzyme-linked immunoassay (ELIZA) method (1/10, normal range: up to 1/10). Due to the incomplete response to steroids, the steroid pulse was repeated three weeks later, but she developed covid-19, her weakness worsened, and then she was unable to walk. The patient was referred to Sina Hospital for further evaluation with a possible diagnosis of Neuromyelitis Optica Spectrum Disorder (NMOSD). MRI of the brain and spinal cord was performed again with gadolinium, which showed LETM with dorsal subpial longitudinal enhancement with trident sign on axial cuts (Fig. 1). According to MRI of the spinal cord, she was diagnosed as a suspected case of Neuro-Sarcoidosis (NS), so the diagnostic workup was done for systemic involvement. Chest high-resolution computed tomography (HRCT) scan imaging was normal. The vasculitis tests were negative except for the high titer of the SSA antibody. The patient's clinical symptoms and minor salivary glands biopsy were negative for Sjogren's syndrome. Whole-body fluorodeoxyglucose positron emission tomography (FDG-PET) was negative. A lung perfusion scan was negative. Because of the progression of paraparesis, cyclophosphamide was started. After receiving cyclophosphamide pulse (1gr monthly for 3 months) and rituximab and oral steroid she was able to stand without walking after 4 months. She was readmitted to be treated with infliximab as a refractory NS if recommended by a rheumatologist.

During admission to the neurology department, a cardiac consult was requested due to the patient’s nonspecific symptoms like atypical chest pain regardless of exertion, shortness of breath at function class of II and dizziness. She was assessed by history taking, ECG, and echocardiography at first. None of the complaints such as orthopnea, paroxysmal nocturnal dyspnea, palpitation or history of syncope or faint were registered.

**Fig. 2:**
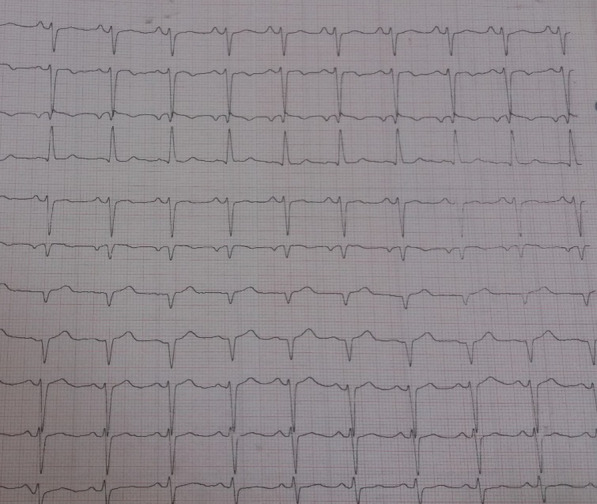
Electrocardiogram showed normal sinus rhythm, left axis deviation, Left Anterior Hemi Block (LAHB), and poor R Wave progression. *LAHB* Left Anterior Hemi Block

In the physical exam, she had normal systolic and diastolic blood pressure, regular pulse, and weak radial and femoral pulse pressure. Neither a heart murmur nor lung crackle was detected. Electrocardiogram showed normal sinus rhythm, left axis deviation, left anterior hemi block (LAHB), and poor R Wave progression (Fig. 2).

Cardiac biomarkers like highly sensitive troponin and Pro–B-type Natriuretic Peptide (Pro-BNP) were in the normal ranges High-sensitivity Troponin (HsTrop<0.0 ng/ml) and (Pro_BNP <100 pg/mL).

Echocardiography findings revealed mild dilated LV (Left Ventricle) but moderate LV systolic dysfunction with positive regional wall motion abnormality in the territories of the anterior wall including the base of anterior, anteroseptal, anteroapical, apical, septoapical, and thinning and fibrotic changes of myocardial wall in these regions. Also, neither LV clot, significant valvular regurgitation, mass nor vegetation were found in transthoracic echocardiography. The right ventricle wall had normal size with preserved systolic dysfunction. Diastolic dysfunction was registered in grade III. Mild pulmonary hypertension was detected for this patient with Pulmonary Arterial Pressure (PAP) 38mmHg. There was no pericardial effusion or changes in the pericardium matrix. The Inferior Vena Cava (IVC) flow and size were normal (Table 1).

**Table 1:** Echocardiographic parameters

LV size (mm)	58_End diastolic_/33_End Systolic_
RV diameter (mm)	28
LA(mm)	35
RA(mm)	21
Diastolic function	Grade III, E 105cm.s, A 23cm/s, DT 75msec, septal Ĕ 5cm/s, lateral Ĕ7cm/s
LV systolic function	35-40%
Reginal Wall Motion Abnormality	Anteroseptal, anteroapical, apical, septoapical
TR gradient	32mmHg
Pulmonary Atrial Pressure	36-38mmHg
Pericaldium	Normal thickness, no effusion

*cm/s* centimeters per second, *DT* Deceleration Time, *LA* Left Atrial, *LV* Left Ventricle, *mm* millimeter, *mmHg* mmHg: millimeter of mercury high, *RA* Right Atrial, *RV* Right Ventricle, *TDI* Peak velocity in early diastole of tricuspid annulus, *TR* Tricuspid Regurgitation

The cardiac team had planned for both cardiac MRI and coronary angiography. Coronary angiography from the radial artery was unsuccessful and the patient did not allow to do it via femoral site.

**Fig. 3:**
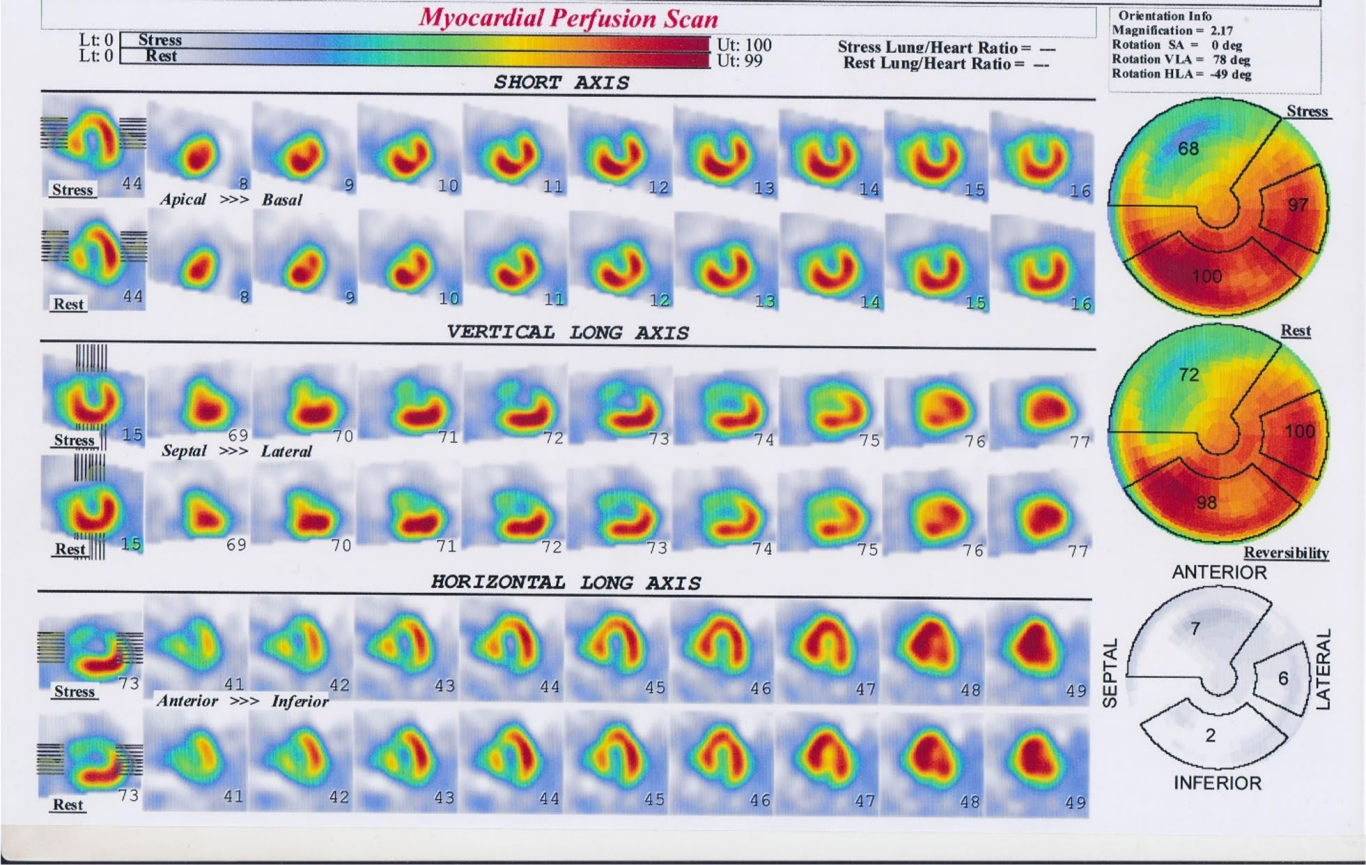
Myocardial Perfusion Imaging Single-Photon Emission Computed Tomography (MPI-SPECT) as illustrated has revealed fixed lesion of the anterior and anteroseptal walls. *MPI-SPECT* Myocardial Perfusion Imaging Single-Photon Emission Computed Tomography

So, we had to confirm the etiology of ischemia by myocardial perfusion scan. Myocardial Perfusion Imaging Single-Photon Emission Computed Tomography (MPI-SPECT), as illustrated in (Fig. 3) has revealed fixed lesions of the anterior and anteroseptal walls.

**Fig. 4:**
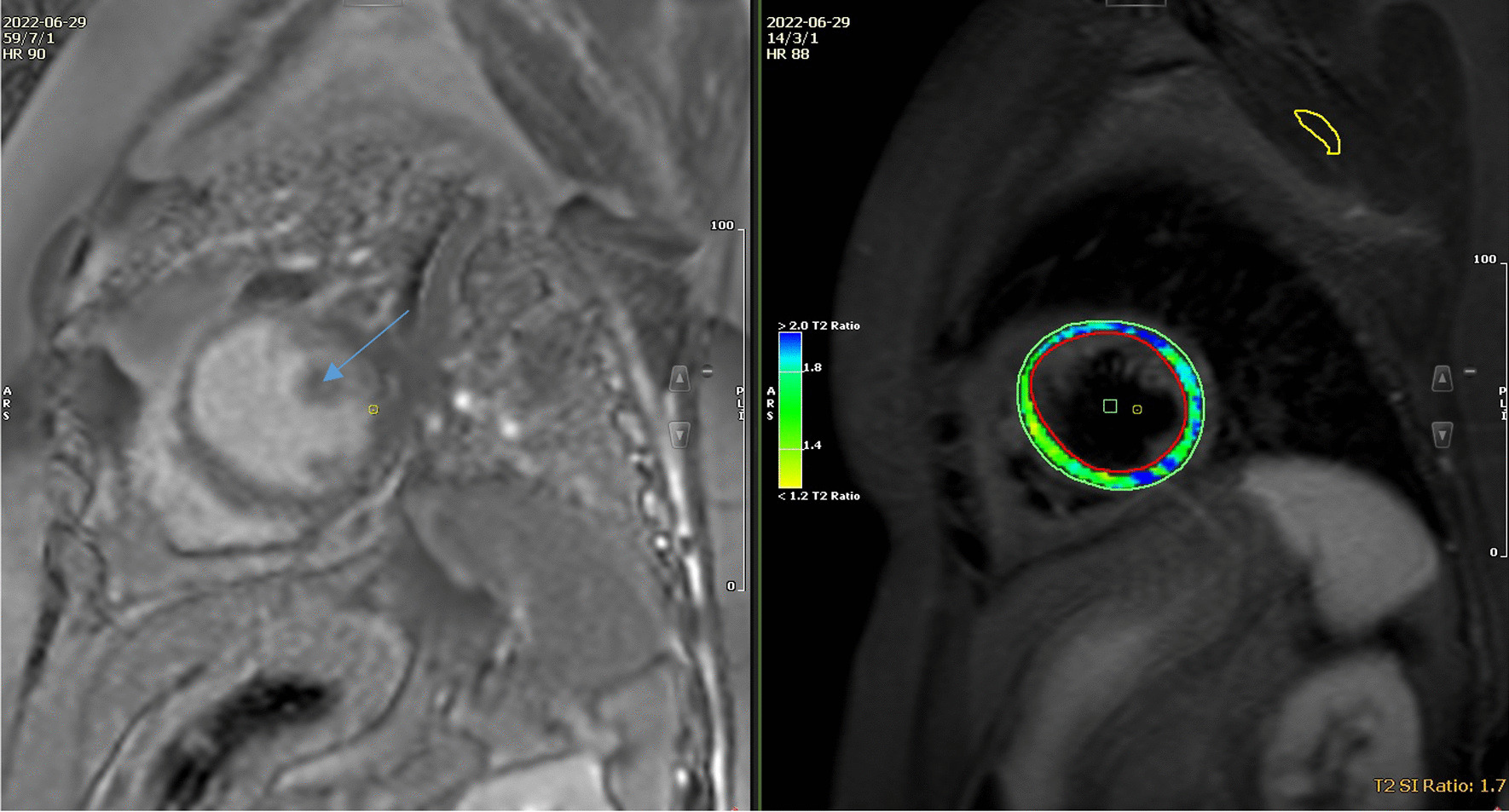
Short Axis STIR images show multifocal edematous changes in anterior, lateral and inferior walls of LV basal to mid segments which are compatible with sarcoid granulomas (arrow)

**Fig. 5:**
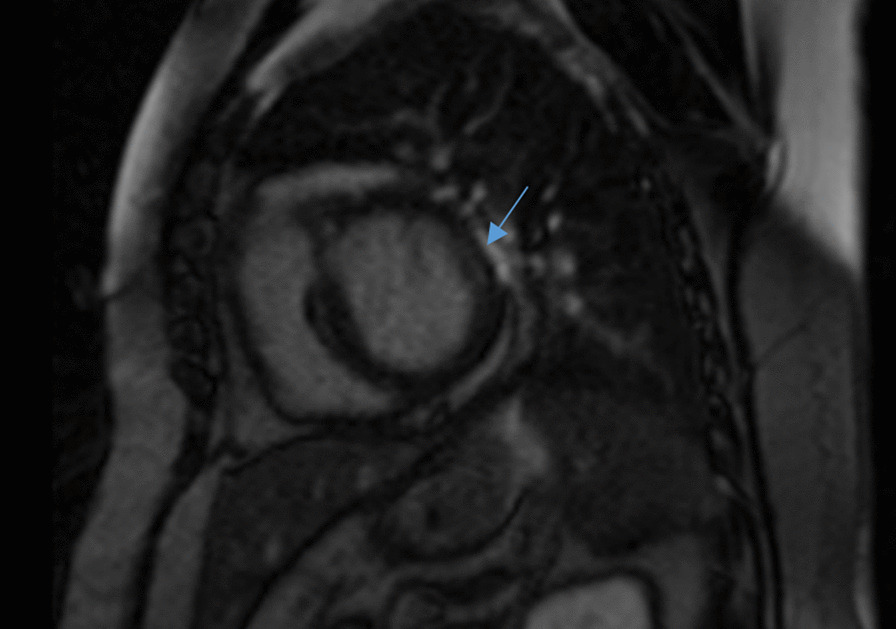
Short axis late gadolinium enhancement images demonstrate large burden of myocardial loss and thinning in basal LV segment with intact endocardium and also patchy mid mural to subepicardial enhancement in mid part of septum (arrow). *LV* Left Ventricle

A cardiac MRI was performed at Rajaee Heart Center, Tehran, Iran. 4-chamber Cine images demonstrate global severe hypo-kinesia associated with septal wall dys-synchrony more prominent in the basal segment as shown in Figures 4, 5.

Short axis STIR images shows multifocal edematous changes in anterior, lateral and inferior walls of LV basal to mid segments which are compatible with Sarcoid granulomas (Fig. 4).

Short axis late gadolinium enhancement images demonstrate a large burden of myocardial loss and thinning in the basal LV segment with intact endocardium and also patchy mid mural to subepicardial enhancement in mid part of the septum (Fig. 5).

24 ECG Holter monitoring showed only rare PAC (Premature Atrial Complex) and PVC (Premature Ventricular Complex) without any episode of VT, or advanced block or pause. Furthermore, we have decided to treat the patient with carvedilol, spironolactone, atorvastatin, and aspirin and follow her with symptoms and serial echocardiography and electrocardiogram.

After cardiac evaluation, the patient was diagnosed with sarcoidosis with two organs involvement and infliximab was started for the patient because of very specific imaging findings in both cardiac and spinal cord MRI. Two months after infliximab she was able to walk with a walker about 20 meters.

## Discussion

Sarcoidosis is a multisystem cell-mediated inflammatory disorder in which the formation of non- or limited necrotizing epithelioid cell granuloma due to unknown antigenic causes is pathologic hallmark of the disease [1]. Nervous system (central or peripheral system) involvement is seen in 5 to 10% of sarcoidosis patients defined as NS, which is the first manifestation of the disease in more than half of them [4]. In 31% of patients with NS presentations, there is systemic involvement from the beginning, and in others, it develops during the disease [1, 4]. Cranial nerve involvement, including the optic and facial nerves, is the most common manifestation, and myelopathy or myelitis accounts for about one-fifth of cases [4].

Myelopathy caused by sarcoidosis, could be in the differential diagnosis of other inflammatory diseases such as NMOSD [5]. Longitudinally extensive transverse myelitis (LETM) (≥3 vertebral segments on MRI) is one of the diagnostic features of NMOSD but should be considered after excluding other differential diagnoses [6].

The same type of involvement is usually seen in NS patients with spinal cord myelopathy, but some key diagnostic findings in clinical and MRI can differentiate between these two diseases [4].

Usually, the onset of paraparesis in sarcoidosis patients is gradual and chronic as in our case, and its onset is less acute and subacute [5, 6]. Subpial enhancement in the ventral or especially in the dorsal region is more common in sarcoidosis but is not exclusive [6]. The combination of the central and dorsal channels involvement creates a special appearance in axial sequences MRI, which is very diagnostic for sarcoidosis and is called trident sign [7, 8]. Taking a biopsy sample is particularly difficult to perform in cases of spinal cord involvement, this imaging view can be very helpful in the need to carefully screen patients for systemic involvement [5, 8, 9].

Therefore, in suspicious cases, investigation of systemic signs and symptoms in clinical and para-clinical workup can help to accurately diagnose these cases [1, 5].

Interpretation of the ELISA Aquaporin-4- IgG (AQp4) antibody test should be done with caution because of high false positive results in medium or low titers and the positivity of this test should be confirmed by the CBA method. One case was previously reported with a misdiagnosis due to the ELISA test [9]. Lung tissue involvement and bilateral hilar lymphadenopathy are seen in more than 90% of patients, high-resolution computed tomography (HRCT) scan imaging could be very diagnostic [1, 10]. But patient's HRCT result was negative.

It is possible to identify asymptomatic occult granulomatous inflammation by using fluorodeoxyglucose positron emission tomography (FDG-PET), which shows increased glucose uptake of active inflammation [10]. Therefore, if a chest CT scan is negative, the FDG PET scan is a more sensitive method for evaluation of extensive lymphadenopathy or disease-related activity in other organs such as cardiac or bones [10]. In our patient, whole-body FDG PET also was negative.

Systemic sarcoidosis has cardiac involvement in up to 25% of patients' autopsy. Cardiac involvement portends a worse prognosis [2, 3]. CS can range from asymptomatic to ventricular tachycardia (VT), high-grade atrioventricular (AV) block, or heart failure and rarely sudden cardiac death as a first presentation of CS due to brady- or tachyarrhythmias [2].

The sensitivity of endomyocardial biopsy is hindered by the myocardial tissue's patchy involvement, thereby limiting its effectiveness as a diagnostic method. Therefore, it is recommended to perform a cardiac MRI or FDG PET scan [3].

According to the diagnostic criteria of CS in addition to histological or clinical diagnosis of extra-cardiac sarcoidosis, two or more of the five major criteria below or one of the major criteria and ≥ two of the minor criteria are satisfied to confirm probable CS [2].

Major cardiac involvement criteria include:

-  High-grade AV block or fatal ventricular arrhythmia
- Basal thinning of the ventricular septum or abnormal ventricular wall anatomy
- Abnormally high uptake with 67Ga citrate or 18F-FDG-PET
- Depressed ejection fraction of the LV (< 50%)
- Delayed enhancement on gadolinium-enhanced MRI
- Minor criteria include
- ECG: ventricular arrhythmias, BBB, axis deviation, or abnormal Q waves
- Perfusion defects by myocardial perfusion scintigraphy
- EMB (endomyocardial biopsy): monocyte infiltration and moderate or severe myocardial interstitial fibrosis

In our patient, there are two major criteria of reduced Left Ventricular Ejection Fraction (LVEF) and delayed enhancement on gadolinium-enhanced MRI and also two minor criteria of LAHB in electrocardiogram and perfusion defects by myocardial perfusion scintigraphy. So, the satisfied CS was confirmed for her. As it is described in our patient, STIR images show multifocal edematous changes in the anterior, lateral and the inferior walls of LV basal compatible with sarcoid granulomas.

Because of the high basal glucose metabolism of the cardiac tissue, the FDG-PET scan may have a diagnostic pitfall in showing inflammatory areas with a high metabolism. Therefore, the preparation of the patient before the FDG-PET scans with different methods such as a proper diet can reduce this defect in the evaluation of cardiac sarcoidosis [3, 10].

Deciding the type and duration of treatment in NS and CS patients is currently challenging [11, 12].

In patients with spinal sarcoidosis or symptomatic cardiac involvement, treatment with oral steroids and simultaneous initiation of an immunosuppressive drug such as Methotrexate Azathioprine or Mycophenolate Mofetil is recommended. In refractory cases, it is recommended to start infliximab but its use is contraindicated in cases of heart failure [11, 12].

## Conclusion

This case report indicated that Sarcoidosis may present with variety of signs and symptoms associated with wide range of radiological findings which needs to be promptly recognized and carefully treated.

## Data Availability

The datasets used during the current study are available from the corresponding author on reasonable request.

## Abbreviations

AQp4: Aquaporin-4- IgG
AV: Atrio-Ventricular
CS: Cardiac sarcoidosis
ECG: Electro-Cardio-Gram
EMB: Endo-myocardial biopsy
FDG-PET: Fluoro-Deoxyglucose Positron Emission Tomography
Ga67: Gallium-67
gr: Gram
HsTrop: High-sensitivity Troponin
HRCT: High-Resolution Computed Tomography
HRS: Heart Rhythm Society
JMHW: Japanese Ministry of Health & Welfare
IVC: Inferior Vena Cava
LAHB: Left Anterior Hemi Block
LETM: Longitudinally Extensive Transverse Myelitis
LV: Left Ventricle
LVEF: Left Ventricular Ejection Fraction
mg: Milligram
mmHg: Millimeter of mercury high
MRI: Magnetic Resonance Imaging
ng/ml: Nano-grams per milliliter
NMOSD: Neuromyelitis Optica Spectrum Disorder
NS: Neuro-Sarcoidosis
PAC: Premature Atrial Complex
PAP: Pulmonary Arterial Pressure
pg/mL: Pico-grams per milliliter
Pro-BNP: Pro–B-type Natriuretic Peptide
PVC: Premature Ventricular Complex
SSA: Anti-Sjogren's Syndrome
STIR: Short Tau Inversion Recovery
VT: Ventricular Tachycardia
WASOG: World Association of Sarcoidosis and Other Granulomatous Disorders Sarcoidosis Organ

## References

[1] Stern BJ, Royal W, Gelfand JM, Clifford DB, Tavee J, Pawate S (2018). Definition and consensus diagnostic criteria for neurosarcoidosis: from the neurosarcoidosis consortium consensus group. JAMA Neurol.

[2] Mankad P, Mitchell B, Birnie D, Kron J (2019). Cardiac sarcoidosis. Curr Cardiol Rep.

[3] Manabe O, Oyama-Manabe N, Aikawa T, Tsuneta S, Tamaki N (2021). Advances in diagnostic imaging for cardiac sarcoidosis. J Clin Med.

[4] Bradshaw MJ, Pawate S, Koth LL, Cho TA, Gelfand JM (2021). Neurosarcoidosis: pathophysiology, diagnosis, and treatment. Neurol Neuroimmunol Neuroinflamm..

[5] Murphy OC, Salazar-Camelo A, Jimenez JA, Barreras P, Reyes MI, Garcia MA (2020). Clinical and MRI phenotypes of sarcoidosis-associated myelopathy. Neurol Neuroimmunol Neuroinflamm..

[6] Flanagan EP, Kaufmann TJ, Krecke KN, Aksamit AJ, Pittock SJ, Keegan BM (2016). Discriminating long myelitis of neuromyelitis optica from sarcoidosis. Ann Neurol.

[7] Zalewski NL, Krecke KN, Weinshenker BG, Aksamit AJ, Conway BL, McKeon A (2016). Central canal enhancement and the trident sign in spinal cord sarcoidosis. Neurology.

[8] Gibbons E, Whittam D, Jacob A, Huda S (2021). Images of the month 1: Trident sign and neurosarcoidosis. Clin Med.

[9] Jolliffe EA, Keegan BM, Flanagan EP (2018). Trident sign trumps Aquaporin-4-IgG ELISA in diagnostic value in a case of longitudinally extensive transverse myelitis. Mult Scler Relat Disord.

[10] Akaike G, Itani M, Shah H, Ahuja J, Yilmaz Gunes B, Assaker R (2018). PET/CT in the diagnosis and workup of sarcoidosis: focus on atypical manifestations. Radiographics.

[11] Voortman M, Drent M, Baughman RP (2019). Management of neurosarcoidosis: a clinical challenge. Curr Opin Neurol.

[12] Giblin GT, Murphy L, Stewart GC, Desai AS, Di Carli MF, Blankstein R (2021). Cardiac sarcoidosis: When and how to treat inflammation. Cardiac Fail Rev..

